# Single-nucleotide polymorphism discovery by high-throughput sequencing in sorghum

**DOI:** 10.1186/1471-2164-12-352

**Published:** 2011-07-07

**Authors:** James C Nelson, Shichen Wang, Yuye Wu, Xianran Li, Ginny Antony, Frank F White, Jianming Yu

**Affiliations:** 1 Department of Plant Pathology, Kansas State University, 4024 Throckmorton Plant Sciences Center, Manhattan KS 66506, USA; 2 Department of Agronomy, Kansas State University, Manhattan KS 66506, USA

## Abstract

**Background:**

Eight diverse sorghum (*Sorghum bicolor *L. Moench) accessions were subjected to short-read genome sequencing to characterize the distribution of single-nucleotide polymorphisms (SNPs). Two strategies were used for DNA library preparation. Missing SNP genotype data were imputed by local haplotype comparison. The effect of library type and genomic diversity on SNP discovery and imputation are evaluated.

**Results:**

Alignment of eight genome equivalents (6 Gb) to the public reference genome revealed 283,000 SNPs at ≥82% confirmation probability. Sequencing from libraries constructed to limit sequencing to start at defined restriction sites led to genotyping 10-fold more SNPs in all 8 accessions, and correctly imputing 11% more missing data, than from semirandom libraries. The SNP yield advantage of the reduced-representation method was less than expected, since up to one fifth of reads started at noncanonical restriction sites and up to one third of restriction sites predicted *in silico *to yield unique alignments were not sampled at near-saturation. For imputation accuracy, the availability of a genomically similar accession in the germplasm panel was more important than panel size or sequencing coverage.

**Conclusions:**

A sequence quantity of 3 million 50-base reads per accession using a *Bsr*FI library would conservatively provide satisfactory genotyping of 96,000 sorghum SNPs. For most reliable SNP-genotype imputation in shallowly sequenced genomes, germplasm panels should consist of pairs or groups of genomically similar entries. These results may help in designing strategies for economical genotyping-by-sequencing of large numbers of plant accessions.

## Background

Among the major world crops, sorghum (*Sorghum bicolor *L. Moench) presents one of the more attractive targets for rapid genetic improvement: high abiotic-stress tolerance, the efficient C_4 _photosynthesis pathway, high genetic diversity, and an available genome sequence. A grass belonging to the Andropogonaceae, sorghum ranks fifth among world crops in weight of grain produced [1]. While in developed countries its grain and silage are used mainly for cattle feed, in many African, Asian, and Latin American countries sorghum grain is a staple of human nutrition and the rest of the plant provides feed and fuel. The special importance of sorghum for subsistence farmers in arid and infertile lands is due to its higher level of tolerance to drought and to waterlogging and its more frugal use of soil nitrogen fertilizer than other cereal crops [2]. Of late the crop has attracted interest for bioenergy production owing to these agronomic advantages, its extravagant biomass production potential, and the availability of sweet sorghums with high sugar concentration in the stem [3].

The relatively small genome size of sorghum (736 Mb, about twice that of rice), high genetic diversity, diploidy, minimal level of gene duplication facilitating functional assignment, and ability to serve as a model for crops with the C_4 _carbon-fixing metabolism such as maize, pearl millet, and sugar cane, justified its choice as the third plant species to have its genome sequenced. An 8x draft sequence of cultivar BTx623 was released in 2007 [4].

Prospects for molecular-genetic improvement of sorghum are based on long research into its phenotypic and genetic diversity. As a focus for diversity study, the U.S. sorghum community has created a panel of 378 grain-sorghum accessions from diverse geographic and climatic regions, including 230 lines generated via backcross conversion of diverse African landraces to short, daylength-insensitive U.S. lines [5], and 148 elite grain or forage cultivars and other accessions of genetic or historical importance. The panel accessions were genotyped with 47 single-sequence-repeat (SSR) markers [6] for a preliminary assessment of diversity patterns of relevance to association mapping. In a second study [7] the panel was genotyped at selected loci surrounding two dwarfing genes for fine-mapping by association.

The primary target in variation studies has come to be single-nucleotide polymorphisms or SNPs. While these can be mined from existing sequence databases [8], recent advances in high-throughput DNA sequencing (HTS) [9-11] have made possible the direct characterization, at steadily decreasing cost, of all the nucleotide-level variation present between individuals of a species, only a fraction of which is accessible to conventional genetic markers. Current technologies yield reads of 25 to more than 400 bases, in gigabase quantities per sequencing run. SNPs are identified by comparison of sequence reads between two or more accessions. Availability of a reference genome sequence both speeds this resequencing operation and allows genomic mapping of polymorphisms. When a reference sequence is not available, a common practice is to assemble reads into contigs and align all reads against them [12-16].

For relatively small genomes, SNP discovery and even genotyping [17,18] can be accomplished by whole-genome sequencing. For large genomes, whether or not a reference sequence is available, much of the sequence thus collected will be repetitive and of little value for polymorphism discovery, while the gene space will be only shallowly sampled. "Reduced-representation" or "genomic-reduction" approaches seek to reduce the extent of the sampled region and to enhance the sampling of gene regions. They have taken several forms including EST sequencing [19-21], methylation digestion [22], and restriction-fragment-size-based sampling [12-16,23-29]. The restriction-site associated DNA (RAD) approach [30,31] involves constructing a sequencing library from only genomic DNA fragments whose 5' ends abut the recognition site of a selected restriction enzyme. The choice of enzyme allows tuning the numbers of sites assayed, while read labeling by attachment of short DNA identifying sequences known as multiplex identifiers (MIDs), or "bar codes" allows tuning the depth of coverage according to the number of individuals pooled in the same sequencing reaction.

The throughput and economy of HTS renders increasingly attractive the prospect of genomewide SNP genotyping without the development of individual SNP assays or dedicated hybridization chips. Recent applications of "genotyping by sequencing" (GBS) produced massive SNP genotype sets for hundreds of rice accessions [17] and a 150-line biparental mapping population [32] via a strategy relying on shallow multiplex sequencing of bar-coded DNAs. The incomplete SNP haplotypes of the component individuals were then completed by computational imputation of missing genotypes and of recombination breakpoints based on haplotype information from the nonmissing data. Most current SNP imputation methods focus on methods applied in high-density human SNP-typing studies, where a reference panel may be available but where phase inference is a source of error [33]. In the two large plant studies cited above, genotype reconstruction from partial data was accurate to above 95%, in the one partly because in inbred individuals phasing is obviated, and in the other owing to the defined linkage disequilibrium of the biparental population. But in the still-young field of plant GBS, SNP genotype inference has not been evaluated in smaller accession panels, and reference panels are not yet available.

Validation of putative SNPs generated in a small germplasm panel is often incomplete because of the expense of exhaustive confirmatory assays. Unless sequencing is done to high coverage, commonly a small set of SNP candidates is selected for confirmation by Sanger sequencing or hybridization assays. An average of 5× coverage per maize accession yielded a confirmation rate of 95% of 92 tested SNPs [34], while 45× coverage in rice yielded in two different genomes 88 and 95% confirmation of 731 SNPs via a designed SNP array [35]. In soybean [26] confirmation rates were only 72 to 85%, with the higher rate, corresponding to more stringent test criteria, reducing by fivefold the number of SNPs called. A false-discovery rate of 15% was reported for another maize SNP-discovery experiment [13], but this study was conducted without a reference genome and faced the problem of computationally distinguishing paralogous from allelic SNPs in an ancestral tetraploid. Another soybean study [36], using several SNP prefiltering criteria including 80× sequencing of another reference genome, reported false-positive and -negative rates of 1.8 and 3.5%. The latter useful statistic, quantifying the proportion of SNPs missed by a method, is not always reported; confirmation rates of up to 97% in soybean and rice were reported by [29], but without mention of the accompanying false-negative rate. As a rule confirmation rates depend heavily on the initial filtering algorithm used to declare SNP candidates, since many unfiltered SNP candidates are sequencing errors.

The expected density of SNPs in plant genomes is relevant to their utility for further genetic studies. In the few plant species subjected to large-scale SNP exploration by HTS, SNP density has been reported to range from 107 bases per SNP in rice [32] to 200 in maize [13], 370 in soybean genes [21], and 500 in *Arabidopsis *[18]. But density will vary with genome region, depth of sequencing coverage, and choice of sampled accessions as well as with the breeding system of the species.

The aim of the study reported here was the sequencing, to sufficient depth for discovery of a set of 100,000 to 250,000 SNPs, of a representative set of grain-sorghum accessions from the panel described above. In the process it became of interest to examine the parameters that affect the potential efficiency of reduced multiplex sequencing and genotype imputation. The longer-term purpose is to generate a foundation for wider allelic exploration of sorghum germplasm resources and to develop a finer-scale picture of the genetic variation present in this and other sorghum germplasm collections that will support the search for alleles of value for sorghum breeding.

## Results

### Sequencing summary

**Figure 1:**
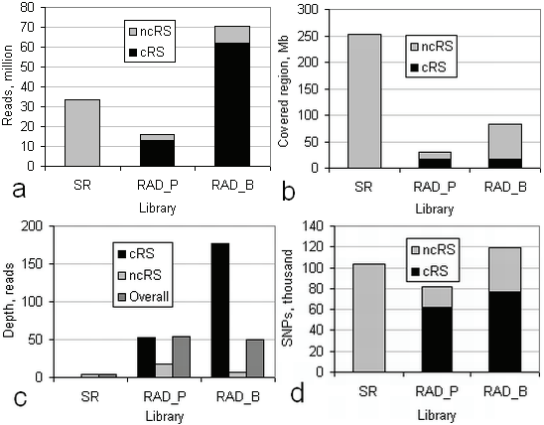
**Sequence and SNP characteristics from semirandom and RAD sorghum libraries**. a) Total uniquely aligning reads; b) length of genome spanned; c) average coverage depth of reads; d) total SNPs in reads. cRS: canonical; ncRS: noncanonical restriction sites at 3' ends of reads. SR: semirandom library.

From the three DNA libraries 247M reads of length varying from 32 to 76 bases were obtained (Table 1). These yielded about 8 genome equivalents (6 Gb) of uniquely alignable sequence (Figure 1a) covering slightly more than a third of the sorghum genome (Figure 1b). The RAD libraries gave about a 15-fold better coverage depth in the sampled genomic region than the semirandom (SR) library (Figure 1c), based on equal numbers of uniquely aligning reads accepted after quality filtering. The generally higher quality of reads from the RAD than from the SR library was not considered in this comparison, since library construction and sequencing were done at different times by different laboratories.

**Table 1:** Sequencing summary for three sorghum libraries

Statistic	SR*	RAD_P	RAD_B
Sequencing lanes	7	5	4
Length of reads after trimming (nt)	32	35, 76	56
Total reads, × 10^6^	103	42	96
% remaining after quality filter	40	50	97
% uniquely aligned (UA) reads	33	38	73
Total length of UA reads, Gb	1.1	1.1	3.6

*Semirandom

The RAD library unique alignments were more enriched for genic regions than those from the SR library (*p *<< 10^-10 ^by Z test for comparison of two proportions). While about 15% of the genome is annotated as genic, 38% of RAD_P and 34% of RAD_B reads came from genic regions, in contrast to 28% of SR-library reads.

### Restriction-site coverage by reads from RAD libraries

Cumulative coverage of restriction sites by reads approached plateaus at about 73% (*Pst*I) and 57% (*Bsr*FI) of *in silico *restriction sites (Additional file 1, Figure S1). Since about 86% of reads sampled *in silico *from *Pst*I or *Bsr*FI restriction sites could be aligned uniquely to the genome, it appears that 13% to 29% of sites were not sampled by RAD library preparation. The *in-silico *study showed that the nonsampled restriction sites were not disproportionately associated with short fragment lengths (results not shown), hinting at a biological rather than a library-preparation cause.

The RAD technology also sampled other genomic sites than intended. About 12% of unique alignments of RAD_B and 19% of RAD_P reads were to positions other than the canonical restriction sites (cRS) of the respective enzyme (Figure 1a). The alignment positions of these reads originating at noncanonical restriction sites (ncRS reads) appeared to be randomly distributed with respect to the cRS. However, more than half of ncRS in RAD_B reads fell within 500 bases of a cRS, and the genomic regions covered by the two kinds of reads overlapped by up to 40%, depending on the accession and RAD library. Of the > 4000 different 6 b sequences at the starts of the ncRS-reads in RAD_B, 27% showed pattern YCCGGR, resembling the canonical *Bsr*FI recognition sequence RCCGGY. No evident rule governed the accession-coverage frequencies of specific ncRS sequences. The contrasts between accession-coverage frequencies for the two kinds of read origin were very similar for the two RAD libraries (Figure 1d), with large numbers of ncRS sites represented in only one accession but many cRS sites represented in all of the accessions.

### Nucleotide variant characteristics

Over all three libraries, 283 K candidate SNPs passed the Novoalign SNP filter, in contrast to 155 K SNPs called from SOAP2 alignments based on a simple filter requiring ≥ 6 alternative calls with average alternative-allele base-quality score ≥ 20. In the Sanger validation sequencing, the 137 SNPs confirmed in the reads covering the validation set included 106 (79%) of the 134 SOAP2 and 123 (82%) of the 148 Novoalign candidates. The recall proportion or fraction of true SNPs called correctly (1 - false-negative rate) thus favored the Novoalign criteria (123/137 = 0.91) over the simple filter (106/137 = 0.77). "SNP" will in this report accordingly denote candidate SNPs with a Novoalign score of at least 20 and thus at least 82% confirmation probability. This level of uncertainty should be borne in mind in assessing quantitative SNP characteristics to be described below, though we suggest that it has little influence on bulk genome- or accession-related distributional properties of SNPs and their alleles. The inverse relationship between confirmation rate and Novoalign quality threshold as applied to the entire SNP set is shown in Additional file 2, Figure S2. No heterozygous SNPs were identified in the validation set.

**Figure 2:**
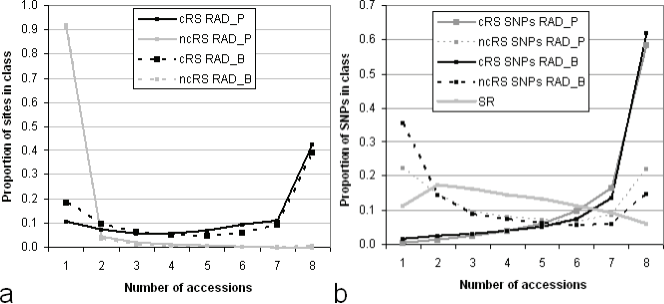
**Accession coverage frequencies of alignment start sites and SNPs of canonical and noncanonical reads in sorghum sequencing libraries**. Accession coverage refers to the number of sorghum accessions in which a read a) originated from the start site, b) covered the SNP. cRS: canonical restriction site; ncRS: noncanonical restriction site: SR: semirandom library

In RAD libraries, cRS and ncRS reads showed very different characteristics, as would be expected from the semirandomly positioned genomic origin of the latter. Of SNPs identified in RAD sequences, up to one third were in ncRS reads (Figure 2a). For the RAD libraries, the accession coverage of SNPs in cRS and ncRS reads showed (Figure 2b) the same extreme difference as seen for the read start sites, with the cRS heavily weighted towards coverage of all eight accessions and the ncRS towards occurrence mainly in individual accessions. The apparent exception to the latter rule seen for ncRS reads in the figure is discussed later. Reads from the SR library also gave little common coverage in multiple accessions (Figure 2b), showing that the *Hpa*II methylation-sensitive digestion and size separation used as a preparation step did not confer detectable complexity reduction for read sampling and SNP discovery

The effectiveness of the RAD approach in focusing sequencing on a limited number of sites was reflected in the result that only half of the SNPs from the SR library but 80% of those from RAD libraries were covered by reads in at least 5 of 8 accessions, even with the lower sequencing coverage in the RAD libraries shown in Figure 1b. Corresponding to this difference, the RAD libraries showed only 20% (RAD_P) and 28% (RAD_B) missing SNP genotype calls in comparison with 50% from the SR library. Noteworthy in the genotype frequencies over the 283 K SNPs from the union of the RAD and SR libraries (Additional file 3, Figure S3) are the relatively high heterozygosity in *Sb*2 (BTx430), *Sb*3, *Sb*7, and *Sb*8, the higher representation of *Sb*2 mentioned above, and the near-identity of *Sb*1 (nominally BTx623) to the BTx623 reference.

**Figure 3:**
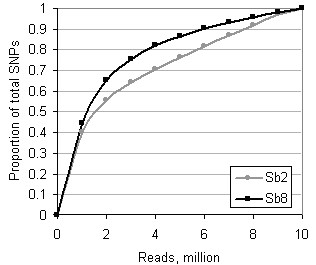
**Relationship of SNP discovery to RAD sequencing depth**. Calculated for the RAD_B library for all chromosomes of accessions *Sb*2 and *Sb*8

A further consequence of RAD enrichment was the more rapid saturation of SNP genotypes with increasing numbers of reads (Figure 3). In test accessions *Sb*2 and *Sb*8, a random sample of around half of the reads produced nearly 80% of the SNP genotypes called in the full data.

Indels accounted for 17% of the polymorphism types. Of the 59,000 indels of length 1 to 13 bp (in the shorter reads of the RAD_B library indels of only up to length 9 were called), 80% were 1 to 3 bp long. Indel density followed SNP density over the genome (*r *= 0.9 based on 100-Kb sliding windows; not shown). The log distribution of indels by accession frequency formed a linear figure (Additional file 4, Figure S4) matching that of SNP alternative-allele frequency.

### Genome distribution and functional characteristics of nucleotide variation

Polymorphism showed strong spatial patterning across sorghum chromosomes (Additional file 5, Figure S5), with highest polymorphism focused at ends of chromosomes and lowest in regions annotated as repetitive. The densities of SNPs and indels were similar in all genomic feature classes (Additional file 6, Figure S6). However, the size distribution of indels was highly nonuniform across feature classes, and favored multiples of 3 in exons with respect to intergenic regions (Additional file 7, Figure S7).

The ratios of non-synonymous to synonymous SNPs varied from 0.81 to 0.97 across the libraries, and that of transition to transversion for candidate SNPs from 1.3 to 1.5.

Of 60 K exonic SNPs, 2325 were large-effect SNPs and were present in 5.6% of annotated sorghum genes. Large-effect SNPs were concentrated in families associated with signaling and molecular recognition functions (Additional file 8, Figure S8).

### Accession diversity

**Figure 4:**
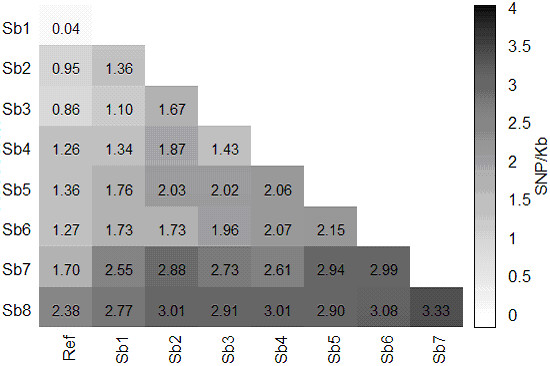
**Pairwise relative SNP density in genomes of eight sorghum accessions and reference accession BTx623**. Densities were derived from SNPs in reads from the RAD_P library.

**Figure 5:**
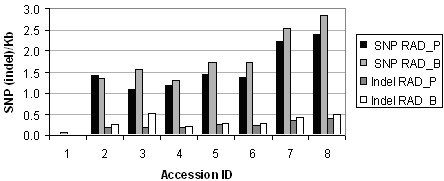
**SNP and indel density by accession in sorghum RAD sequence**. Only reads from canonical restriction sites were used; noncanonical reads show the same trend but lower densities (data not shown).

In terms of pairwise relative SNP density, the noncultivated *propinquum Sb*7 and subspecies *verticilliflorum Sb*8 showed highest divergence from the other accessions, while *Sb*1 showed the expected high concordance with the BTx623 reference (Figures 4, 5). SNP density between accessions was uniformly higher than that between accessions and the reference. Both RAD libraries gave similar diversity statistics, while the SR library showed the same trend but with much lower densities.

**Figure 6:**
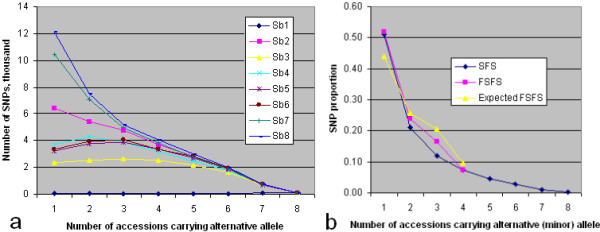
**Allele-frequency distributions of SNPs from the RAD_P sequencing library**. a) Each point for an accession denotes the number of SNPs having the indicated alternative (non-BTx623)-allele frequency in which that accession carried or was imputed the alternative allele. It is computed from original plus imputed SNP genotype data; the plot from only original complete data is essentially identical. b) Unfolded- (SFS) and folded- (FSFS) site-frequency-spectrum plots showing distribution of alternative (SFS) or minor (*i.e. *whether BTx623 or non-BTx623) (FSFS) allele by numbers of accessions. Expected FSFS: the FSFS expected under the infinite-sites, no-selection model with random mating; see text.

About half of all SNPs were represented by a single alternative allele (Figure 6). Of these half were accounted for by the noncultivated accessions *Sb*7 and *Sb*8, making one fourth of the SNPs due solely to these two accessions. The corresponding skewing towards rare alleles of the site-frequency spectrum in comparison with that expected under the infinite-sites mutation model with no selection [37] is not surprising in view of the heterogeneous origins and cultivation histories of the accessions. The near-zero frequency of SNPs with alternative (*i.e. *non-BTx623) alleles in all 8 accessions follows from the rarity of these alleles in *Sb*1.

**Figure 7:**
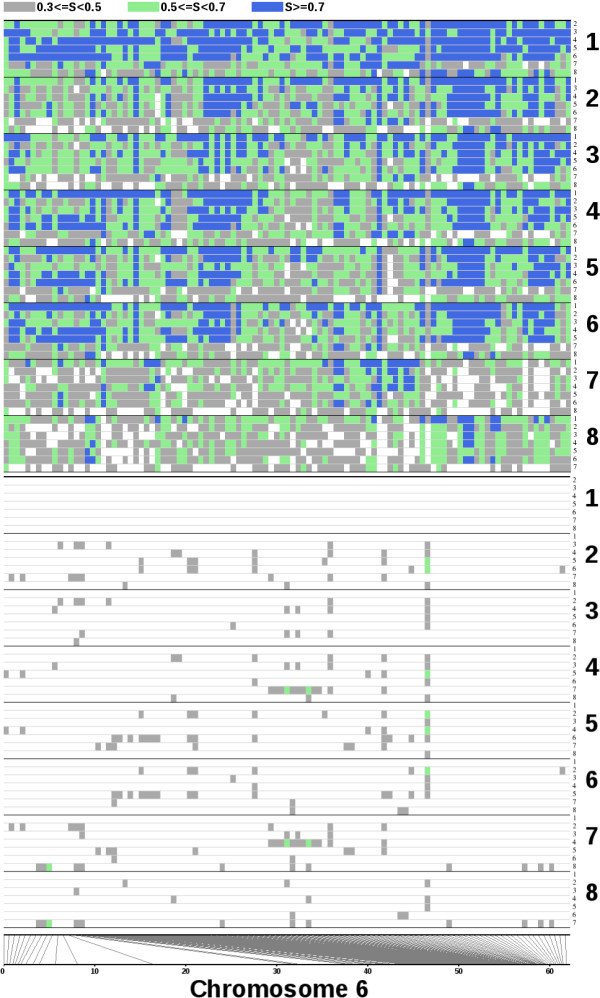
**Pairwise comparison of SNP haplotypes for eight sorghum accessions**. Each main numbered horizontal plot represents one accession. Its seven minor plots represent pairwise genotype comparisons of called SNPs with those in the other seven accessions; in the upper panel only matches of the reference homozygote and in the lower only those of the alternative homozygote are shown. The color coding indicates windows of 50 SNPs with at least 30, 50, or 70% of genotypes in common between the two accessions (coded as S in the legend). Strips at plot bases show correspondence of the SNP windows with the physical map. Accessions 1-8 are BTx623, BTx430, P898012, Segaolane, SC35, SC265, PI653737 (*S. propinquum*), and 12-26 (*S. bicolor *ssp. *verticilliflorum*). Scale at bottom indicates physical positions of SNPs on chromosome. All chromosome plots appear in Figures S9-S18.

Plots of haplotype sharing (Figure 7; Additional file 9, Figure S9; Additional file 10, Figure S10; Additional file 11, Figure S11; Additional file 12, Figure S12; Additional file 13, Figure S13; Additional file 14, Figure S14; Additional file 15, Figure S15; Additional file 16, Figure S16; Additional file 17, Figure S17; Additional file 18, Figure S18) reveal concentrations of shared alleles in certain genomic regions. They also highlight the distinctness of the two noncultivated accessions from the remainder. No indication of continuous tracts of heterozygosity, or of SNPs at which heterozygosity was shared between different accessions, was found.

### SNP genotype imputation and factors affecting accuracy

Average imputation accuracy for any given library depended not on its size but on the completeness and accession balance of its SNP genotype data (Additional file 19, Figure S19). Though the RAD libraries accounted for only 70% of the combined SNPs, accuracy exceeded that in the full set. The cRS SNPs from the RAD_B library could be imputed to 80% accuracy, but the ncRS to only 65%. Imputation accuracy was unchanged even in subsamples of as few as 0.05 of the SNPs.

**Figure 8:**
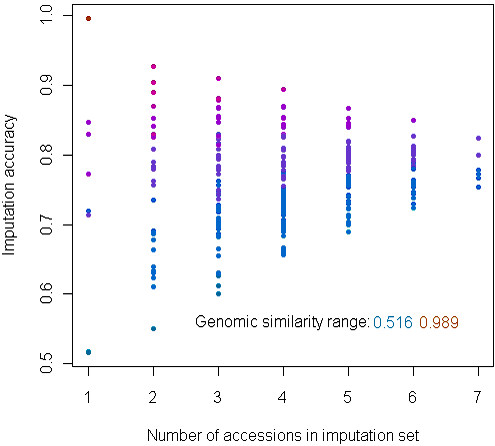
**Relationship of accession group size and genomic similarity with imputation accuracy**. Each point represents a different subset of eight sorghum accessions plus the reference accession, designated as the imputation set. Imputation accuracy describes the proportion of masked loci correctly imputed. Coloring describes the average pairwise genomic similarity in the imputation set, calculated as described in the text.

The window size yielding the highest imputation accuracy was 5 to 15 bases on either side of the target locus. At window size 0, all missing genotypes were imputed as the reference allele for an overall correct-call rate (accuracy) of 0.73, while at the optimum window size, the overall accuracy was 0.79, well above the average similarity of 0.67 between accessions. In agreement with this result, the imputation accuracy for any individual accession exceeded by 0.10 on average the maximum genomic similarity between that accession and any other accession (Additional file 20, Figure S20). Computing imputation accuracy for every subset of accessions showed that accuracy was much more dependent on within-panel similarity than on numbers of accessions in the panel (Figure 8).

## Discussion

### Comparison with prior plant SNP reports

Frequencies of types of nucleotide variation in sorghum were similar to those in other plant species. In six soybean accessions [21] very similar indel fractions and length distributions were reported. Those authors mentioned the higher proportion of 3-multiple indels in exons (compare our Additional file 7, Figure S7), while in six resequenced elite maize inbreds [34] the same phenomenon can be seen if ratios of the coding sequences (CDS) to the intron indel frequencies found in their Table S2 are plotted. Presumably frame-nonshifting are better tolerated than frameshifting indels. SNP transition/transversion ratios of around 1.4 were also similar in this soybean study as in *Arabidopsis *[38]. Nonsynonymous-to-synonymous-substitution ratios of about 0.8 were similar to those in *Arabidopsis *but much lower than the 1.4 reported for soybean by [36] or 1.2 in rice [35,39]. The declining linear trend of log indel or SNP alternative-allele frequency when plotted against the number of accessions in which the indel or SNP appeared has not been examined in other plant-species panels, but likely depends on the choice of germplasm. The original intention of the plot was to suggest gene presence—absence variation (PAV) in a less completely sequenced panel than that of maize in [34], but it implies that such variation is no different from that of individual SNPs, suggesting that PAV may be rarer in our sorghum panel than in the maize panel.

The functional gene families found to be richest in large-effect SNPs and the 6% of genes involved were similar to those reported in *Arabidopsis*, 9% of genes [40], rice, 3% [39], maize [34], and soybean, 10% [36], with enrichment in trafficking, messenger, detoxification, and biotic and abiotic-stress-response functions, notably LRR-containing genes implicated in defense response to pathogens. It was suggested by [40] that such high variation is in accord with rapid gene evolution in response to disease pressure.

The location of highest SNP densities toward sorghum chromosome ends rather than centromeres is similar to that in maize [34] but differs somewhat from that in *Arabidopsis *[40]. This result may trace to the more complete *Arabidopsis *assembly, the lower repetitive-DNA content and its altered genomic distribution, and the difference in genome sampling associated with the array-hybridization approach used for that study. Repetitive sequence near centromeres and telomeres, possibly coupled with missing genomic data owing to the difficulty of assembly in these regions, interferes with unique alignments.

Haplotype comparison is useful for identifying larger genomic tracts differentiating accessions or marking historical introgressions as in rice [39] and maize [34], or for assessing patterns of linkage disequilibrium as in soybean [36]. The patterns of haplotype-block sharing in these eight accessions are clearly not random and suggest that in larger sorghum germplasm panels, reliable identification of correspondences with adaptively important genes will be possible.

### Comparison of RAD with semirandom sequencing for SNP discovery and genotyping

The comparisons between RAD and SR sequencing results presented here do not represent a rigorous side-by-side comparison of two library-preparation methods. Indeed, the RAD approach was adopted only after the inefficiency of the SR approach for our purposes, simultaneous SNP discovery and genotyping, became evident. While our results allow no direct comparison of RAD with other genomic-reduction methods nor of the selected RAD enzymes with the *Hpa*II used for the SR library, they illustrate the advantages of genomic reduction for goals other than enriched gene sequencing.

The adoption of RAD for SNP exploration or genotyping over hybridization or other high-throughput genotyping approaches will depend on the genome, the selection of enzymes, and the laboratory resources available. Semirandom sampling of sorghum sequences led to shallow coverage, by uniquely aligning reads, of one third of the genome, the same proportion as is classed [4] as nonrepetitive. While the RAD approach intensified the sampling of a smaller portion (about 1/50) of the genome, improvement was less than expected for two reasons. First, RAD sampling to near saturation did not cover the available restriction sites to the extent predicted *in silico*. We speculate that systematic cytosine methylation or inefficient cleavage in some restriction sites common to all of the sorghum accessions led to incomplete digestion. Second, one in five to eight RAD reads originated in genomic motifs not matching the canonical recognition sequences of the enzymes. That PCR amplification during library preparation still took place suggests that nonspecific primer binding occurred. While the partially random sequencing that resulted still yielded SNPs, their unsystematic coverage reduced the reliability of SNP-genotype imputation, a key consideration for GBS. To summarize the comparison, 1) about twice as much polymorphism per sequencing outlay was realized by a RAD approach to the same genomic material; 2) the same polymorphisms will be genotyped in other sorghum germplasm sequenced from similarly prepared libraries, in sharp contrast to the situation under SR sequencing; and 3) owing to the much more favorable accession distribution of SNP coverage, imputation is more effective in compensating for partial sequencing. The possible shortcomings of the RAD technology, at least for the two restriction enzymes used here, may be remediable by the use of appropriate digestion buffers (personal communication, R. Nipper) as well as the use of different enzymes and close attention to possible contamination from nonspecific DNAses. Though wasteful, enzyme "leakage" (commonly known as star activity) did not reduce genotyping efficiency to the level of the SR library because of a degree of overlap of the ncRS and cRS reads. This is seen in the U-shaped coverage patterns of Figure 2, where ncRS sites as well as SNPs occur presumably on reads containing cRS sites sampled in all accessions.

### Practical application

While the resampling experiment suggested that halving sequencing coverage of the RAD_B library would sacrifice only one fifth of the SNPs with no loss in imputation accuracy, this estimate is conservative. Practical genotyping will be applied to much larger samples than our seven (not counting *Sb*1- BTx623, for which there would have been no need of genotyping). The worst-imputed genotypes will be in the most divergent accessions, but even in those, local haplotype similarity allows recovery of missing data. The observation that numbers of genomes in the imputation set are much less important in determining imputation accuracy than intergenome similarity implies that an effective strategy for accurate GBS of diverse germplasm panels will be to construct them of groups of at least two members that are predicted to share 80 to 90% SNP similarity. In this way, those loci not sampled in one entry will be accurately imputed if they have been sampled in one of the most similar entries. In our small panel and experimental conditions, the presence of the two wild accessions with similarity neither to each other nor to the cultivated sorghums reduced overall imputation accuracy by 5%. An additional advantage will be realized by the inclusion of multiple reference sequences in the imputation set.

Why did imputation accuracy decline when the SR library was included, even though this library augmented the SNP set by 50%? Regardless of the similarity structure of a panel, the imputation algorithm could assign to a missing SNP in a target accession a genotype only from an accession that possessed one at the target locus. When many of the SNP loci have been defined in only one accession, as in the *Sb*2-skewed read set from the SR library, this accession will usually not be the one most similar to the target accession. Since about half of the SNPs in this set were identified in *Sb*2, this accession and the reference were the only accessions available for imputation at many of the SNPs. The resulting imputation error rate was governed by their average genomewide similarity to the other accessions (Figure 8).

Some estimates of the genetic diversity in our panel may be made by modest extrapolation. The common pairwise genomic coverage achieved by RAD sequencing revealed about 50,000 polymorphisms between each accession pair. Extending the calculated pairwise SNP density to the approximately one third of the sorghum genome that is uniquely alignable suggests that *Sb*2 differs from BTx623 at around 250,000 and *Sb*8 at nearly 600,000 SNPs. Interestingly, by this calculation our *Sb*1, nominally identical to BTx623, differs from that accession at 10,000 SNPs, of which 8200 are expected to be genuine. The proportions of this polymorphism attributable to sequencing or assembly error versus real variation in this accession are unknown, but similar or greater SNP divergence between nominally identical inbred accessions is not novel [29,41].

The above estimates of polymorphism are conservative. The Novoalign algorithm, giving false positives in 18% of calls made at the adopted threshold, failed to call 9% of the confirmed SNPs in the validation set, with 3 of the 12 recall failures due to insufficient coverage depth. Though the SAMtools algorithm gave SNP calls of higher precision and recall than simple depth and quality rules, it is likely that with the high simultaneous coverage afforded by restriction-mediated enrichment, its predictions could be refined by combined evaluation of all accessions for a SNP.

The imputation algorithm we applied, though shown by its developers to be competitive with (and much faster than) more elaborate algorithms, was not designed for the shallow genotyping intensity we contemplate. In a larger set of accessions, imputation rates are likely to be improved by procedures modeling the local haplotype neighborhood of the target accession and not only its haplotype similarity to other accessions. It may also be expected that, as in human diversity studies, reference panels genotyped for many SNPs not necessarily shared by the research set come into use for sorghum and other crops.

## Conclusions

Our focus in this study has been to clarify parameters likely to govern deep and wide genotyping of plants for applied as well as basic-research purposes. Areas of future research interest suggested by our findings might be: the long- and short-term evolutionary bases for the haplotype patterns observed in our germplasm panel; the reasons for the incomplete sampling of genomic restriction sites and their possible functional basis; the gain and loss of larger genomic tracts, from one germplasm accession to the next, than the short indels we have characterized; and efficient algorithms for SNP-genotype completion in the presence of large quantities of missing data.

The sorghum SNP and indel data have been deposited in dbSNP under handle JCNLAB_KSU, with accession-number ranges starting at 410962044 (SNPs) and 411578970 (indels).

## Methods

### Genetic material

Eight accessions were selected from the grain-sorghum diversity panel, including BTx623, the publicly sequenced accession; BTx430; P898012; Segaolane; SC35; SC265; PI653737 (*Sorghum propinquum*); and 12-26 (*Sorghum bicolor *ssp. *verticilliflorum*). The first six of these are parents of a set of mapping populations under development for nested association mapping [42]. BTx430 [43] has been widely used as a pollinator parent to produce sorghum hybrids in the United States and is amenable to genetic transformation [44]. Segaolane is a drought-tolerant kafir-type sorghum from Southern Africa [45]. SC35 is drought resistant and has been used as a staygreen trait donor in sorghum breeding programs in the U.S. and Australia. SC265 was included to represent Guinea/caudatum germplasm [6]. PI653737 was obtained from USDA-GRIN and all other accessions were from the sorghum breeding program at Kansas State University. The accessions were grown out in Manhattan in 2005 and 2006 seasons with heads bagged to prevent outcrossing. In the following, the accessions, in the order listed above, will be referred to as *Sb*1 through *Sb*8.

### DNA extraction

For constructing semirandom (as defined below) sequencing libraries, seeds were surface-sterilized, placed on 0.5× Murashige-Skoog [46] medium, and germinated in the dark. Etiolated 8 d old seedlings were powdered after freezing in liquid nitrogen. DNA was extracted from 250 mg of powdered tissue in a modified buffer using 1% polyvinylpyrrolidone as described by [47]. For the RAD libraries, accessions were grown in soil in the greenhouse until panicles began to form in about 40 DAP. After removal of enclosing leaves, immature panicles were placed into tubes and stored at -70°C until DNA extraction by the method of [48].

### Library construction

Semirandom (SR) libraries were prepared by a commercial sequencing center from 3 μg of DNA digested with *Hpa*II according to the protocol of [13] with the modification that fragments 200 to 2000 instead of 100 to 600 bp in length were excised from the 1% agarose gel. We refer to these libraries as "semirandom" relative to the RAD approach; truly random sampling would have employed nonspecific DNA fragmentation, while a more precisely targeted approach would have employed a narrower fragment range. RAD library sets RAD_P (using *Pst*I) and RAD_B (using *Bsr*FI), were constructed by Floragenex, Inc. (Eugene, Oregon, USA), with a protocol similar to that described by [31]. Briefly, genomic DNA (from each sample ~1 μg) was digested for 60 min at 37°C in a 50 μL reaction with 2 units (U) of *Bsr*FI (or 2 μg with 10 U *Pst*I) (New England Biolabs [NEB]) followed by heat inactivation at 80°C for 20 min. Samples were phenol:chloroform extracted, EtOH precipitated and resuspended in 15 μL Qiagen buffer EB. For RAD_B, 2.0 μL of 10 nM P1 (for RAD_P, 4 μL of 100 nM P5), a modified Solexa^© ^adapter (2006 Illumina, Inc., all rights reserved) containing a unique MID (bar code) of 5 bases for RAD_B and 4 for RAD_P, were added to each sample along with 2 μL 10X T4 DNA ligase buffer (Enzymatics, Inc) and 1.0 μL (600 U) T4 DNA ligase enzyme (high concentration, Enzymatics, Inc) and incubated at room temperature (RT) for 20 min. Samples were again heat-inactivated for 10 min at 65°C, randomly sheared with a Bioruptor (Diagenode) to an average size of 500 bp, and run out on a 1.5% agarose (Sigma), 0.5 × TBE gel. DNA 300 bp to 700 bp was isolated using a MinElute Gel Extraction Kit (Qiagen). T4 polymerase (Enzymatics, Inc) was then used to polish the ends of the DNA. Samples were then purified using a Minelute column (Qiagen) and 15 U of Klenow exo- (Enzymatics) was used to add adenine (Fermentas) overhangs on the 3' end of the DNA at 37°C. After subsequent purification, 1 μL of 10 μM P2, a divergent modified Solexa adapter, was ligated to the obtained DNA fragments at 18°C. Samples were again purified and eluted in 12 μL. The eluate was quantified using a Qubit fluorimeter and 5 ng of this product was used in a PCR amplification with 25 μL Phusion Master Mix (NEB), 2.5 μL of 10 μM modified Solexa Amplification primer mix and up to 20.5 μL H_2_O. Phusion PCR settings followed product guidelines (NEB) for a total of 18 (13 for RAD_P) cycles. Samples were gel purified, excising DNA 300-600 bp, and diluted to 10 nM. Four accessions in each RAD_P library and two in each RAD_B library were pooled for sequencing. The three sets of libraries were sequenced by Illumina sequencing-by-synthesis technology with read lengths ranging from 36 to 76. From the RL, 54% of the sequence was from *Sb*2.

### Studies of restriction enzymes for RAD

In the process of selection of an optimal enzyme, the reference genome was digested *in silico *with each of ten methylation-sensitive enzymes. Reads from both strands at each site were extracted and realigned with the genome and the proportion finding a unique best alignment recorded. Fragment sizes were tabulated. For the two enzymes then used to make RAD libraries, the correspondences between the unique-alignment rate and the fragment size were tabulated.

### Sequence processing

Reads were separated by bar code if applicable and trimmed by 4 bases at 3' ends. Reads with average sequence quality at least 25 were retained. Two methods were used to identify candidate SNPs: 1) SOAP v2 [49] was used to align the preprocessed reads to the reference genome sequence, allowing only unique highest-scoring alignments with a maximum of two mismatches. As a simple filter, polymorphic loci with at least 6 alternative reads having average quality score > = 20 were identified as candidate SNPs; 2) Novoalign (http://www.novocraft.com) was used for alignment followed by application of SAMtools (http://samtools.sourceforge.net/) to identify candidate SNPs and indels. Initial parameter values were: minimum coverage depth 3, minimum SNP quality 20, minimum indel quality 50, with other parameters such as window size and nearby SNP and gap density left at their defaults. Average read coverage was computed as total bases sequenced divided by genomic bases covered by at least one read.

### SNP validation

From reads of *Sb*2 (BTx430), 70 fragments comprising more than 35 kb of DNA and showing the highest density of candidate SNPs were selected. This validation sequence contained 1106 SOAP-called unfiltered mismatches, 121 SNPs called by the simple filter, and 148 SNPs called by NovoAlign based on the parameters described above. PCR primers were designed to yield products of length 550 to 650 bases for Sanger sequencing and target sequence fragments were amplified from BTx430 and sequenced in both directions. Fragments were assembled with PolyPhred [50]. Segments of the reference genome sequence aligning to the reads originally showing the mismatches were then aligned with the validation sequence for comparison of called with confirmed mismatches.

### SNP annotation

SNPs were assigned to genomic feature groups and gene classes according to the sorghum genome annotation (http://www.phytozome.net/sorghum). SNPs were classified as synonymous or nonsynonymous by determination of their position in the CDS of genes followed by examination of the effect of codon change on amino-acid identity. Large-effect SNPs were defined as SNPs lying in exons or in splice sites and expected to lead to alteration of protein structure via a shift between sense and nonsense codons or splicing disruption. Large-effect SNP density was calculated for each PFAM protein family for whose members more than 100 kb of the genic region was covered by reads.

### Genomic similarity

For a proportion statistic useful for comparison with genotype-imputation accuracy, pairwise similarity was calculated as the sum, over the *N *SNP positions genotyped in both accessions, of the number of allele identities, ranging from 0 to 2, at each SNP, divided by 2*N*. Because the number of such SNPs at which neither accession carried an alternative allele depended on the other accessions in the panel, a second statistic was calculated for describing population-independent pairwise diversity as SNP density, S_*ij*_/B_*ij*_. Here S_*ij *_is the sum of allele differences at each SNP shared by accessions *i *and *j*, disregarding linkage phase, and B_*ij *_the number of bases (twice the number of base pairs) sequenced in common in the two accessions.

### SNP genotype imputation

Missing SNP genotypes were imputed by a method adapted from [51]. This entailed examining, for each SNP genotype to be imputed in a given accession, the SNP haplotypes in the remaining accessions (including the reference, an option not considered by those authors), choosing the haplotype most similar to that of the target accession across a window surrounding the SNP and containing a nonmissing genotype at the target SNP, and assigning that genotype to the target accession. In this process the few heterozygous loci were set to missing during data encoding and did not enter into the algorithm, since their genotypes could not be imputed nor used for imputation. The proportion of missing genotypes correctly imputed was assessed by application of the algorithm to the genotype data in which in addition to the originally missing data, 5% or 10% of the genotypes were randomly set to missing. Window size was varied from 0 to 100 and a parameter specifying the degree of similarity required between two haplotypes for imputation to be accepted was also added to the original algorithm. As a test of the influence of sample size and intrasample genomic similarity on imputation accuracy, all subsets of size 1 through 7 from the 8 accessions were evaluated for the statistic.

### Modeling effect of RAD sequencing coverage on SNP genotyping efficiency

In an effort to determine the RAD sequencing coverage required for a specified level of SNP genotyping yield, RAD_B reads for *Sb*2 and *Sb*8 were randomly resampled in increments of 1 million reads. To determine the effect of this sampling on imputation accuracy, SNPs were progressively sampled from the entire set based on the proportions predicted by the SNP yield curves and their imputation statistics were tabulated.

## Competing interests

The authors declare that they have no competing interests.

## Authors' contributions

JCN, FFW, and JY conceived the study and participated in its design and coordination. JCN and SW performed all analyses. GA, YW, and XL assisted with DNA extraction and confirmation sequencing. JCN wrote the paper, with contributions from JY, FFW, and SW. All authors read and approved the final manuscript.

## Supplementary Material

Additional file 1 **Cumulative restriction-site coverage from two RAD sequencing libraries in sorghum**. Distinct sites represented at either of two coverage depths were counted for successive samples of randomly ordered reads from all eight accessions. Click here for file

Additional file 2 **Confirmation proportions of candidate sorghum SNPs relative to SNP quality score**. Source data are a set of 70 sequences in accession BTx430 containing 156 SNP candidates with Novoalign quality score at least 20. The figure projects on the entire SNP set the modest improvement in confirmation rate but sharp decline in available SNPs associated with increased SNP-calling stringency. Click here for file

Additional file 3 **Genotype distributions of SNPs from all sorghum sequencing libraries**. RR, AA, AR: reference- and alternative-allele homozygotes and heterozygote. Click here for file

Additional file 4 **Accession coverage frequencies of indels for three sorghum sequencing libraries**. Describes the allele frequencies of these polymorphisms taken over all accessions; compare with Figure 6a for individual accessions. Plot for full SNP set is included to show functional similarity. Log plot is used to show approximately exponential form of allele distributions. Click here for file

Additional file 5 **Distribution of genomic features on chromosomes**. Features were counted in windows of length L kb at 10-kb offsets, where L was 5000 for SNPs, 500 for large-effect SNPs, and 100 for repetitive DNA content. Click here for file

Additional file 6 **SNP and indel density by genomic feature, for all three sorghum sequencing libraries combined**. Only polymorphisms in reads from canonical restriction sites are included. Click here for file

Additional file 7 **Indel length distribution by genomic feature, all libraries**. Only indels in reads from canonical restriction sites were used; others show the same trend but lower densities. Click here for file

Additional file 8 **Large-effect SNP assignment to gene families**. Density of large-effect (LE) SNPs (see text for definition) for all gene families with more than 100 kb of read coverage by the RAD_B library. Shown in the figure are the twenty families with highest density of LE SNPs. Click here for file

Additional file 9 **Pairwise comparison of chromosome-1 SNP haplotypes for eight sorghum accessions**. Each main numbered horizontal plot represents one accession. Its seven minor plots represent pairwise genotype comparison of called SNPs with those in the other seven accessions. Only matches of the reference homozygote are shown. The color coding indicates windows of 50 SNPs with at least 30, 50, or 80% of genotypes in common between the two accessions (coded as S in the legend). Accessions 1-8 are BTx623, BTx430, P898012, Segaolane, SC35, SC265, PI653737 (*S. propinquum*), and 12-26 (*S. bicolor *ssp. *verticilliflorum*). Scale at bottom indicates physical positions of SNPs on chromosome. Click here for file

Additional file 10 **Pairwise comparison of chromosome-2 SNP haplotypes for eight sorghum accessions**. Description is as for Additional file 9. Click here for file

Additional file 11 **Pairwise comparison of chromosome-3 SNP haplotypes for eight sorghum accessions**. Description is as for Additional file 9. Click here for file

Additional file 12 **Pairwise comparison of chromosome-4 SNP haplotypes for eight sorghum accessions**. Description is as for Additional file 9. Click here for file

Additional file 13 **Pairwise comparison of chromosome-5 SNP haplotypes for eight sorghum accessions**. Description is as for Additional file 9. Click here for file

Additional file 14 **Pairwise comparison of chromosome-6 SNP haplotypes for eight sorghum accessions**. Description is as for Additional file 9. Click here for file

Additional file 15 **Pairwise comparison of chromosome-7 SNP haplotypes for eight sorghum accessions**. Description is as for Additional file 9. Click here for file

Additional file 16 **Pairwise comparison of chromosome-8 SNP haplotypes for eight sorghum accessions**. Description is as for Additional file 9. Click here for file

Additional file 17 **Pairwise comparison of chromosome-9 SNP haplotypes for eight sorghum accessions**. Description is as for Additional file 9. Click here for file

Additional file 18 **Pairwise comparison of chromosome-10 SNP haplotypes for eight sorghum accessions**. Description is as for Additional file 9. Click here for file

Additional file 19 **Data completeness and imputation accuracy for SNPs from three sorghum sequencing libraries**. Missing-data proportion for a library is calculated with respect to all SNPs identified in reads from the library. Click here for file

Additional file 20 **Dependence of SNP genotype imputation accuracy in a sorghum accession on its maximum similarity to any of the remaining seven accessions**. Pairwise similarity was defined on the basis of all SNPs assayed in the panel, as described in the text. Click here for file
