# Supplementary figures and images for: Single-nucleotide polymorphism discovery by high-throughput sequencing in sorghum
Source: BMC Genomics. 2011 Jul 7;12:352. doi: 10.1186/1471-2164-12-352 (PMC3146956; doi:10.1186/1471-2164-12-352)

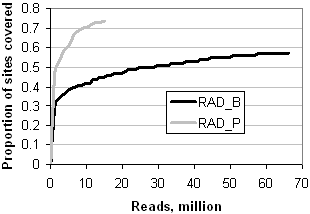

Supplement: Additional file 1 — Cumulative restriction-site coverage from two RAD sequencing libraries in sorghum. Distinct sites represented at either of two coverage depths were counted for successive samples of randomly ordered reads from all eight accessions. [file 1471-2164-12-352-S1.PNG]

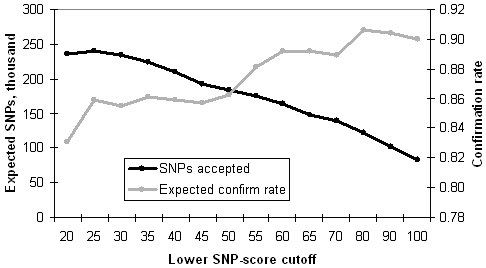

Supplement: Additional file 2 — Confirmation proportions of candidate sorghum SNPs relative to SNP quality score. Source data are a set of 70 sequences in accession BTx430 containing 156 SNP candidates with Novoalign quality score at least 20. The figure projects on the entire SNP set the modest improvement in confirmation rate but sharp decline in available SNPs associated with increased SNP-calling stringency. [file 1471-2164-12-352-S2.PNG]

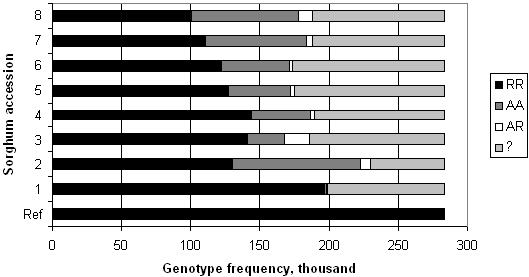

Supplement: Additional file 3 — Genotype distributions of SNPs from all sorghum sequencing libraries. RR, AA, AR: reference- and alternative-allele homozygotes and heterozygote. [file 1471-2164-12-352-S3.PNG]

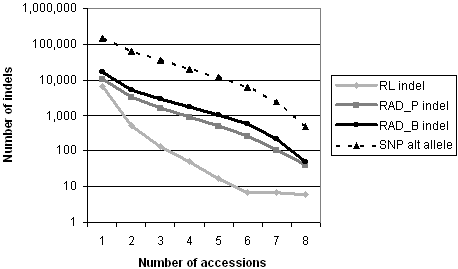

Supplement: Additional file 4 — Accession coverage frequencies of indels for three sorghum sequencing libraries. Describes the allele frequencies of these polymorphisms taken over all accessions; compare with Figure 6a for individual accessions. Plot for full SNP set is included to show functional similarity. Log plot is used to show approximately exponential form of allele distributions. [file 1471-2164-12-352-S4.PNG]

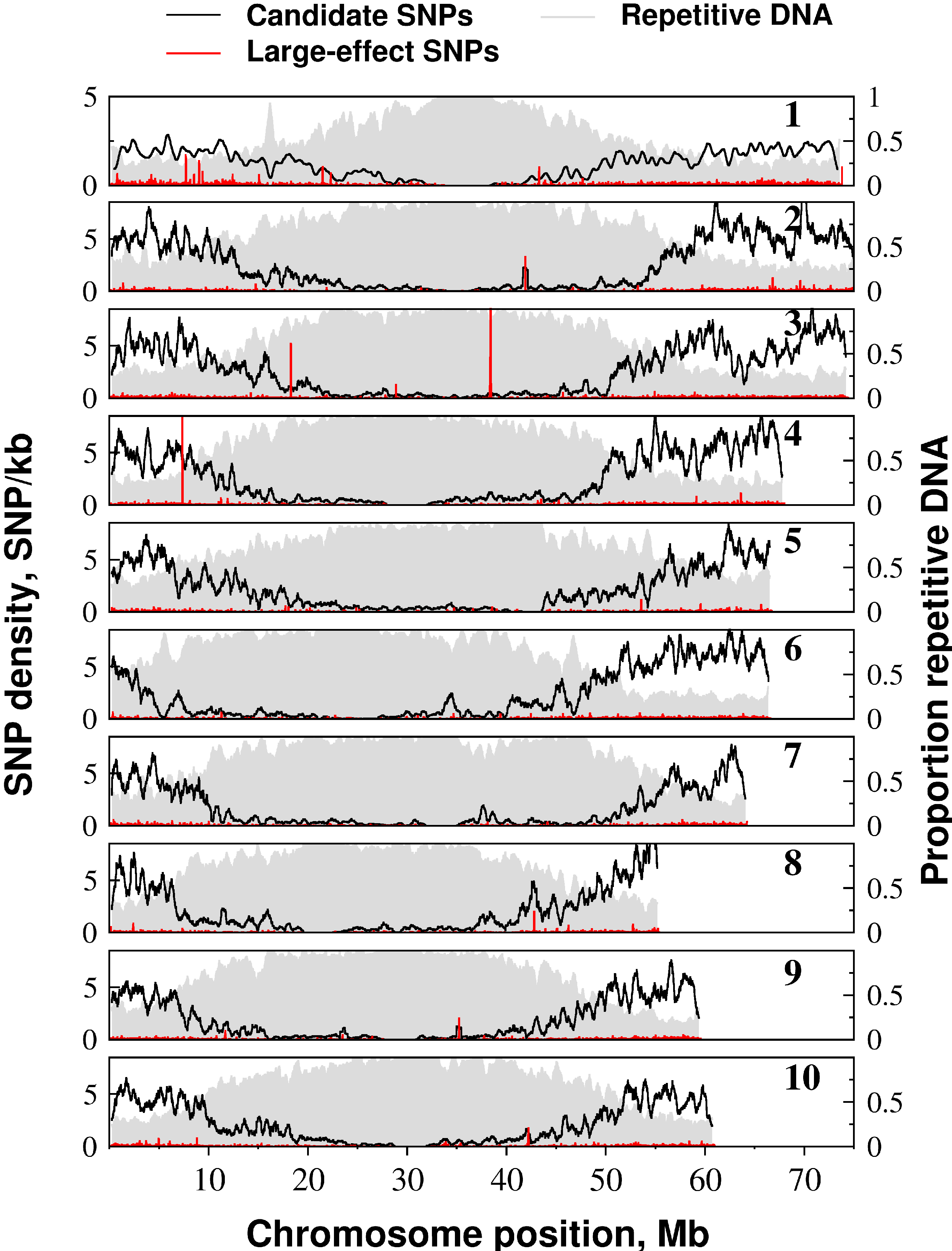

Supplement: Additional file 5 — Distribution of genomic features on chromosomes. Features were counted in windows of length L kb at 10-kb offsets, where L was 5000 for SNPs, 500 for large-effect SNPs, and 100 for repetitive DNA content. [file 1471-2164-12-352-S5.PNG]

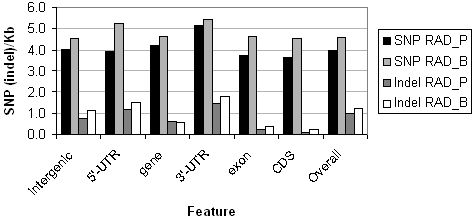

Supplement: Additional file 6 — SNP and indel density by genomic feature, for all three sorghum sequencing libraries combined. Only polymorphisms in reads from canonical restriction sites are included. [file 1471-2164-12-352-S6.PNG]

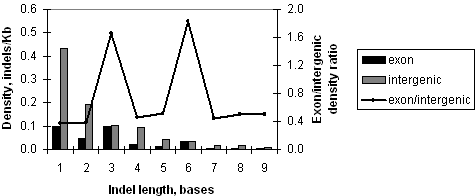

Supplement: Additional file 7 — Indel length distribution by genomic feature, all libraries. Only indels in reads from canonical restriction sites were used; others show the same trend but lower densities. [file 1471-2164-12-352-S7.PNG]

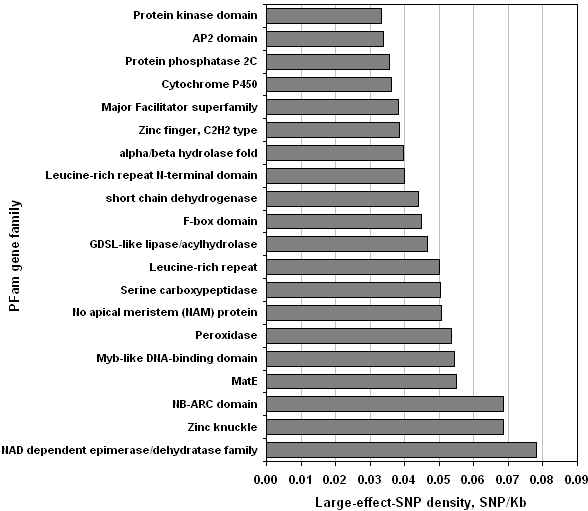

Supplement: Additional file 8 — Large-effect SNP assignment to gene families. Density of large-effect (LE) SNPs (see text for definition) for all gene families with more than 100 kb of read coverage by the RAD_B library. Shown in the figure are the twenty families with highest density of LE SNPs. [file 1471-2164-12-352-S8.PNG]

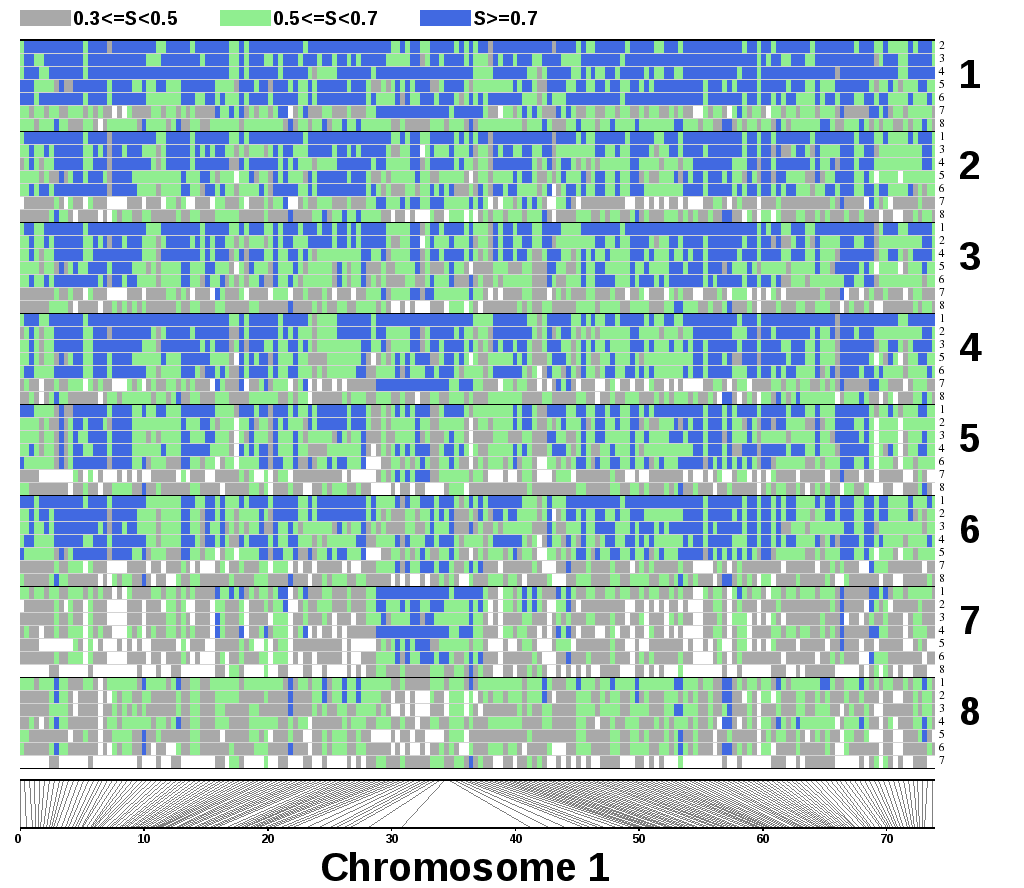

Supplement: Additional file 9 — Pairwise comparison of chromosome-1 SNP haplotypes for eight sorghum accessions. Each main numbered horizontal plot represents one accession. Its seven minor plots represent pairwise genotype comparison of called SNPs with those in the other seven accessions. Only matches of the reference homozygote are shown. The color coding indicates windows of 50 SNPs with at least 30, 50, or 80% of genotypes in common between the two accessions (coded as S in the legend). Accessions 1-8 are BTx623, BTx430, P898012, Segaolane, SC35, SC265, PI653737 (S. propinquum), and 12-26 (S. bicolor ssp. verticilliflorum). Scale at bottom indicates physical positions of SNPs on chromosome. [file 1471-2164-12-352-S9.PNG]

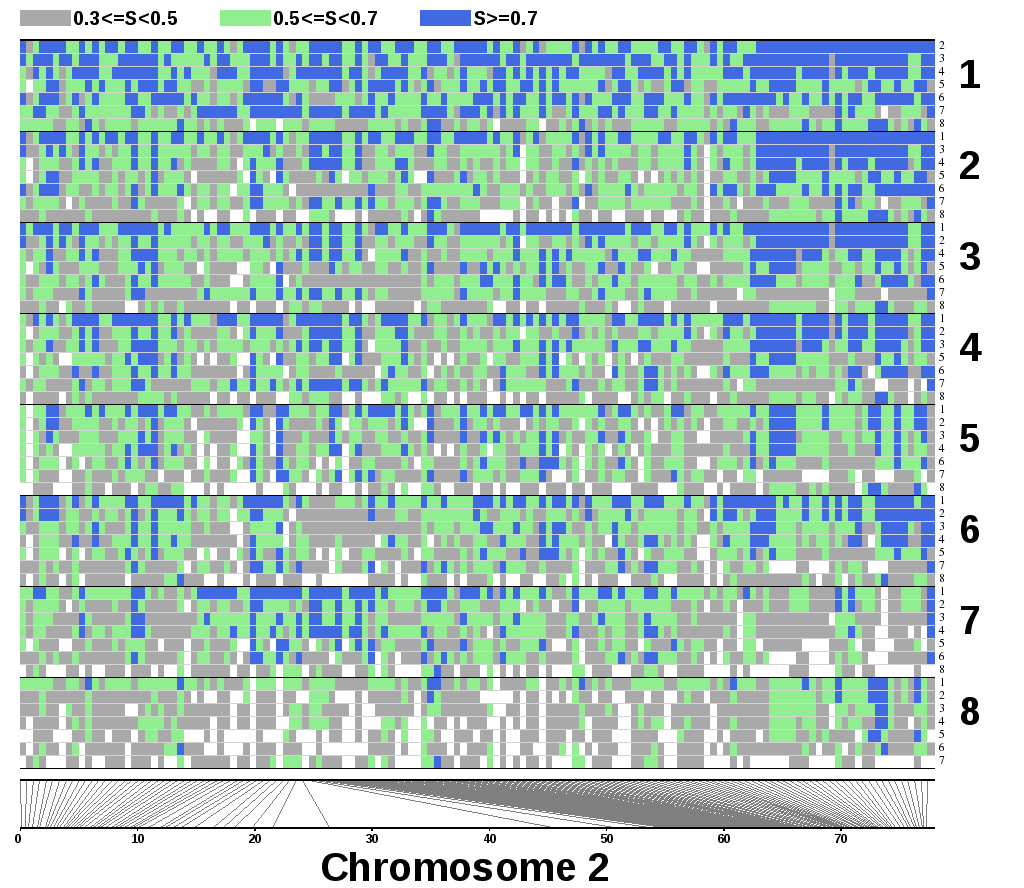

Supplement: Additional file 10 — Pairwise comparison of chromosome-2 SNP haplotypes for eight sorghum accessions. Description is as for Additional file 9. [file 1471-2164-12-352-S10.PNG]

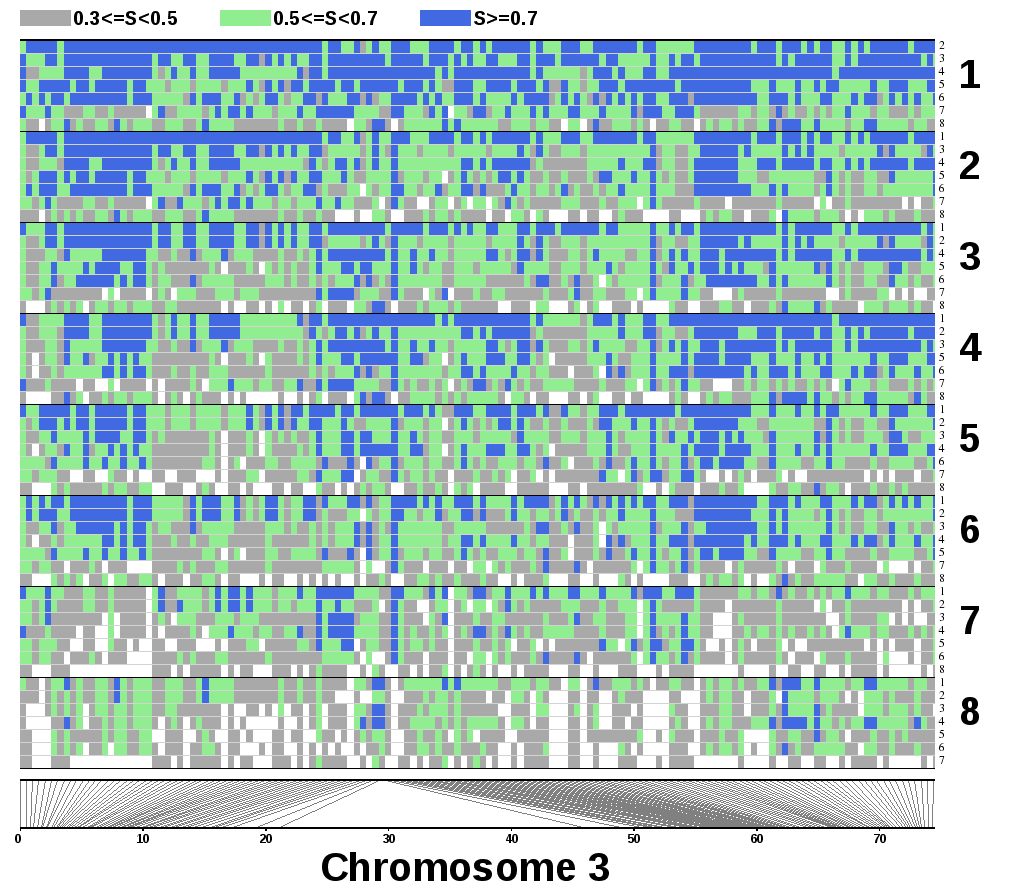

Supplement: Additional file 11 — Pairwise comparison of chromosome-3 SNP haplotypes for eight sorghum accessions. Description is as for Additional file 9. [file 1471-2164-12-352-S11.PNG]

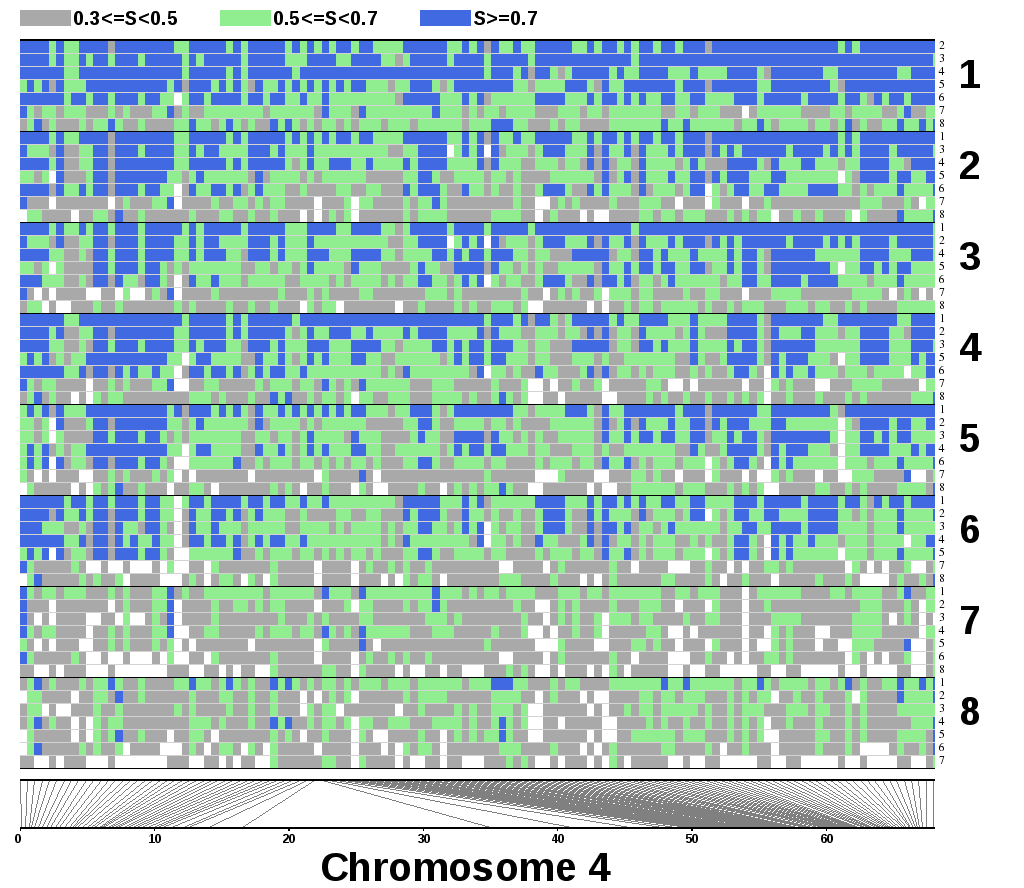

Supplement: Additional file 12 — Pairwise comparison of chromosome-4 SNP haplotypes for eight sorghum accessions. Description is as for Additional file 9. [file 1471-2164-12-352-S12.PNG]

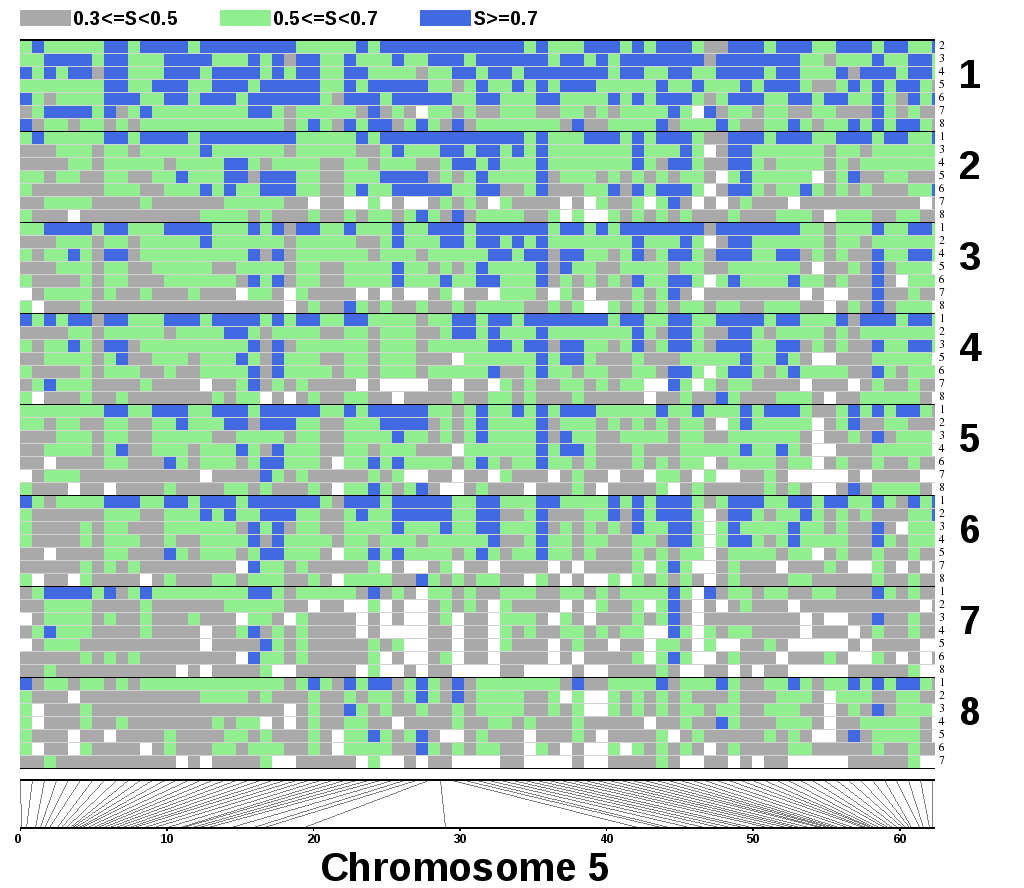

Supplement: Additional file 13 — Pairwise comparison of chromosome-5 SNP haplotypes for eight sorghum accessions. Description is as for Additional file 9. [file 1471-2164-12-352-S13.PNG]

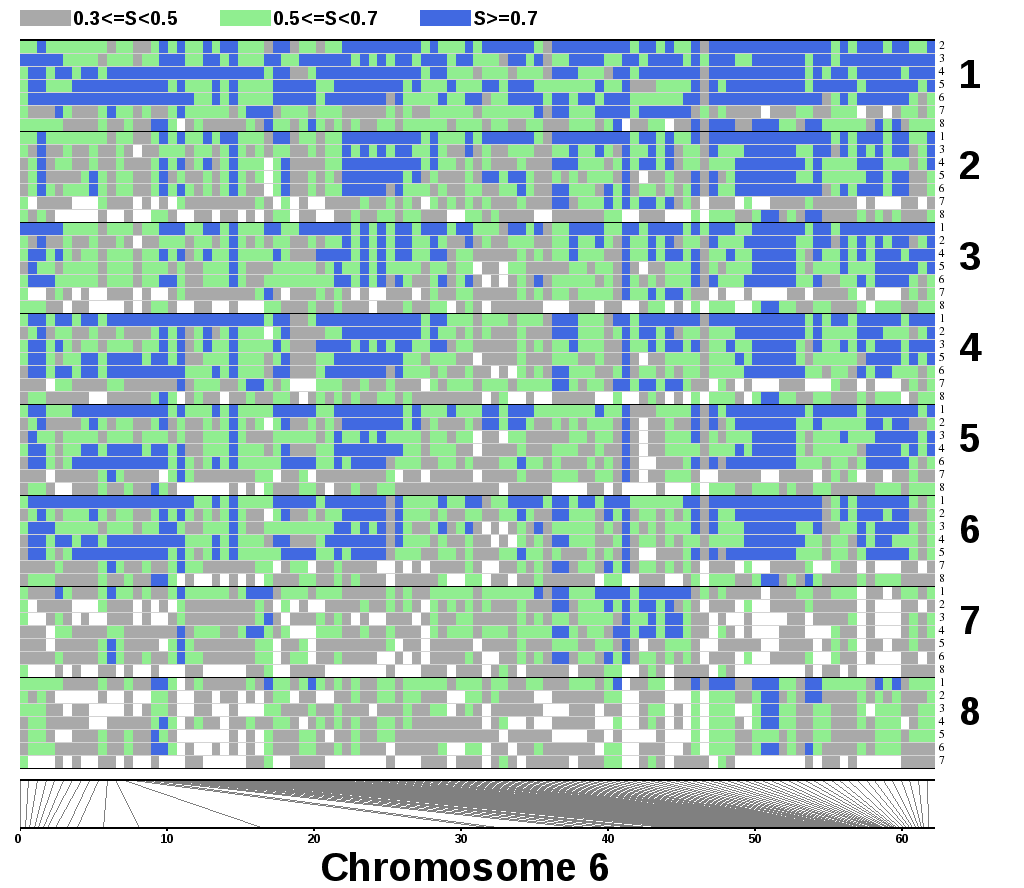

Supplement: Additional file 14 — Pairwise comparison of chromosome-6 SNP haplotypes for eight sorghum accessions. Description is as for Additional file 9. [file 1471-2164-12-352-S14.PNG]

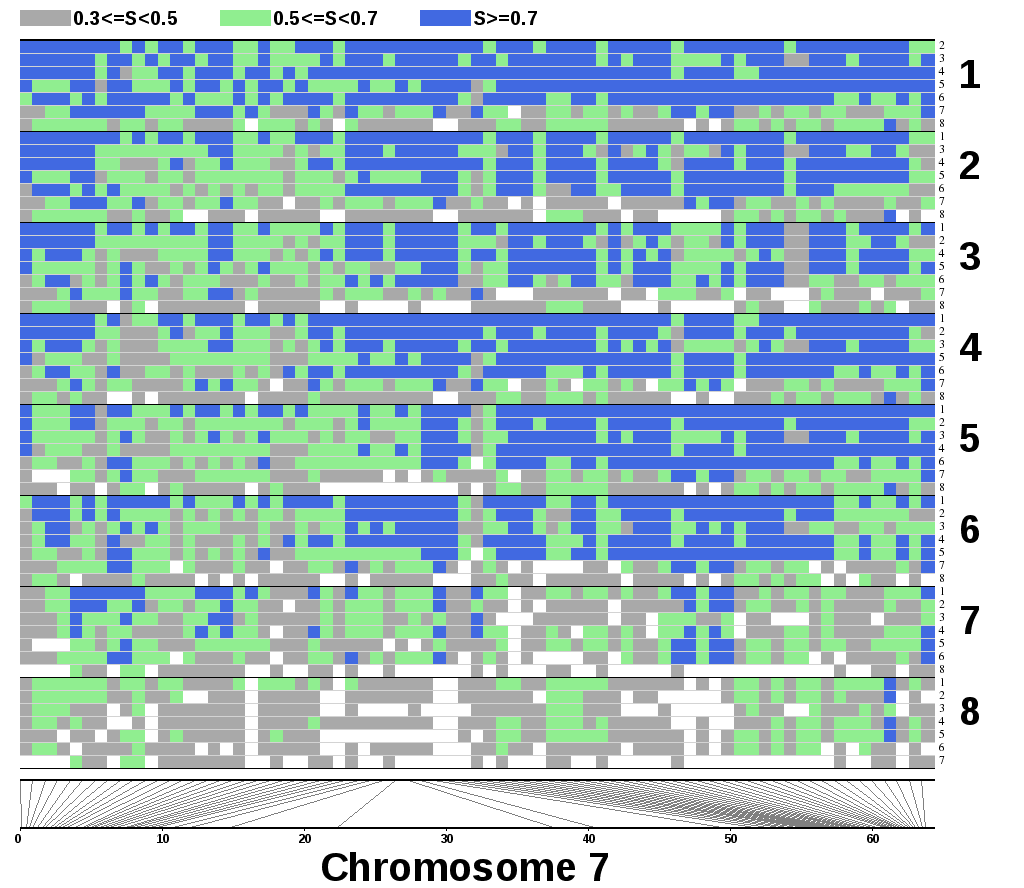

Supplement: Additional file 15 — Pairwise comparison of chromosome-7 SNP haplotypes for eight sorghum accessions. Description is as for Additional file 9. [file 1471-2164-12-352-S15.PNG]

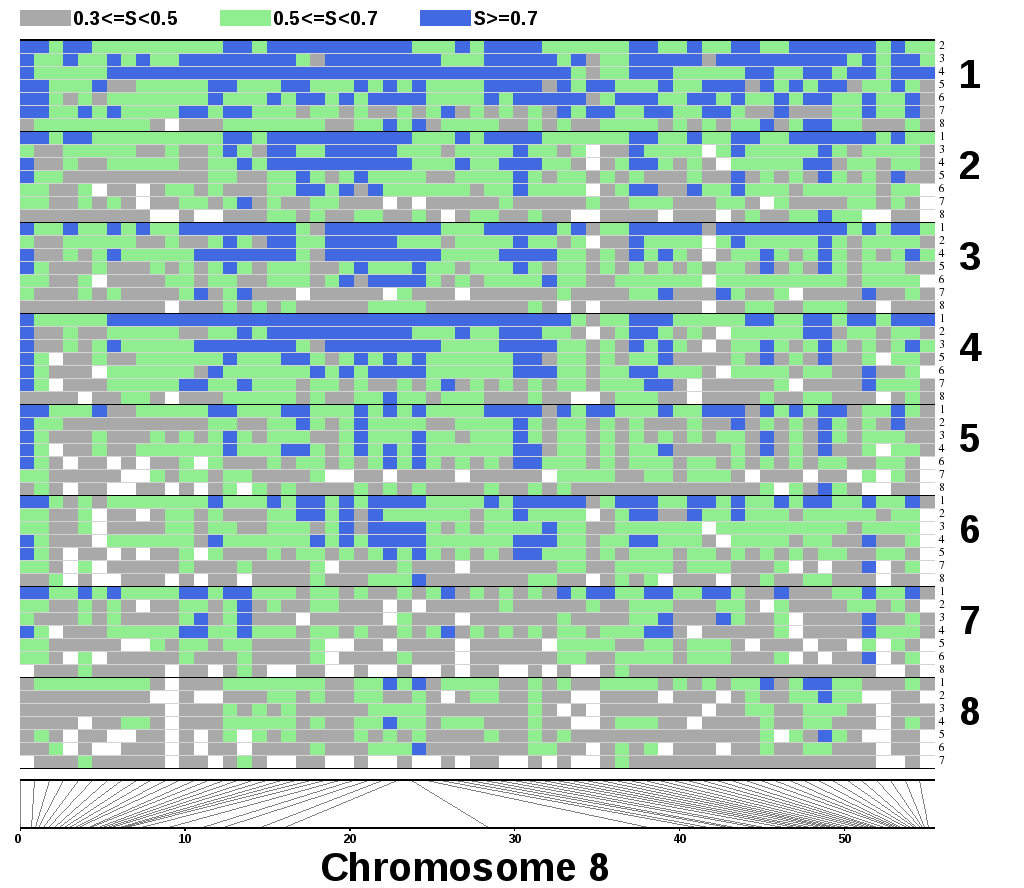

Supplement: Additional file 16 — Pairwise comparison of chromosome-8 SNP haplotypes for eight sorghum accessions. Description is as for Additional file 9. [file 1471-2164-12-352-S16.PNG]

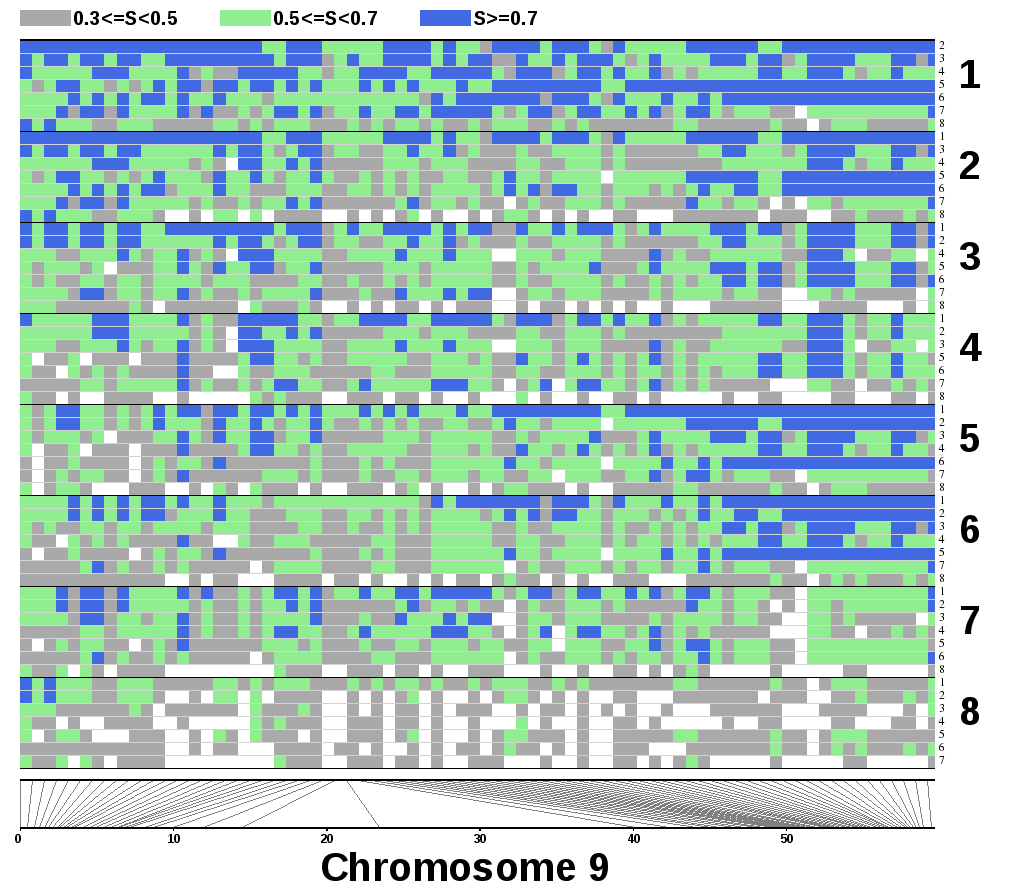

Supplement: Additional file 17 — Pairwise comparison of chromosome-9 SNP haplotypes for eight sorghum accessions. Description is as for Additional file 9. [file 1471-2164-12-352-S17.PNG]

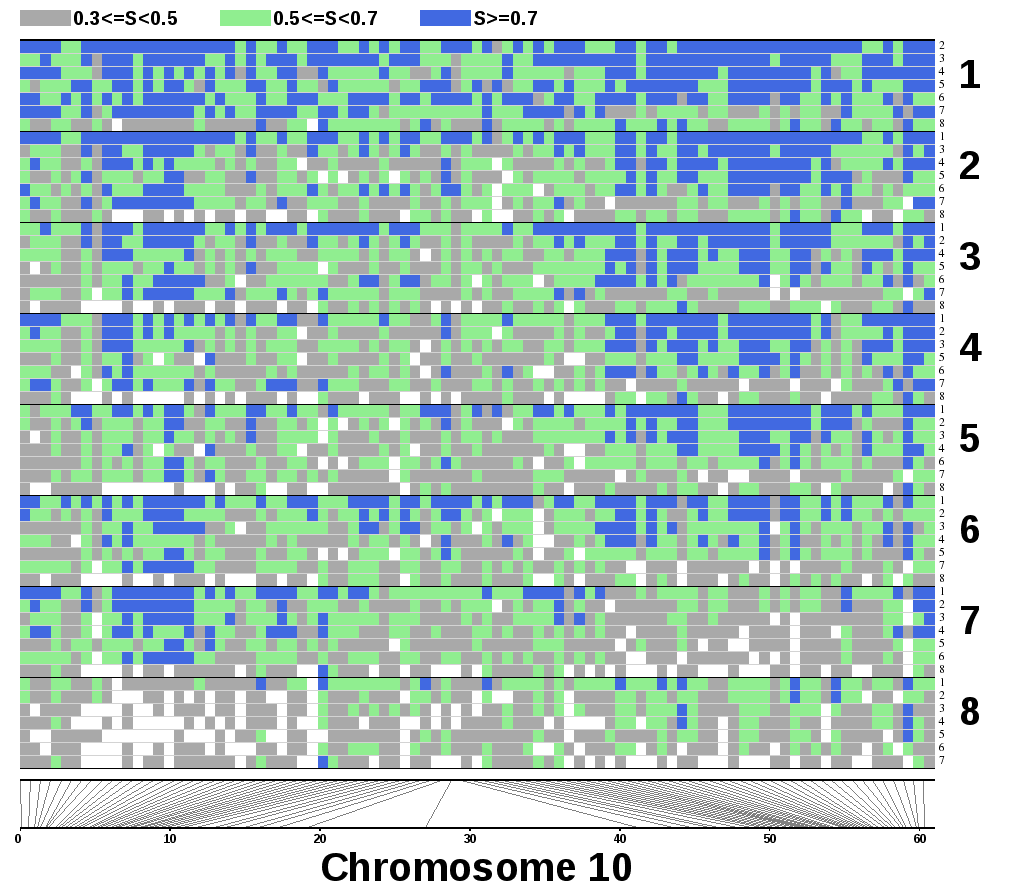

Supplement: Additional file 18 — Pairwise comparison of chromosome-10 SNP haplotypes for eight sorghum accessions. Description is as for Additional file 9. [file 1471-2164-12-352-S18.PNG]

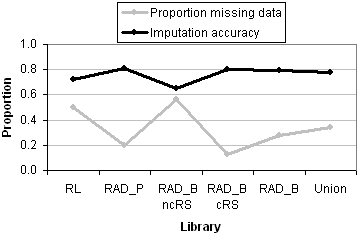

Supplement: Additional file 19 — Data completeness and imputation accuracy for SNPs from three sorghum sequencing libraries. Missing-data proportion for a library is calculated with respect to all SNPs identified in reads from the library. [file 1471-2164-12-352-S19.PNG]

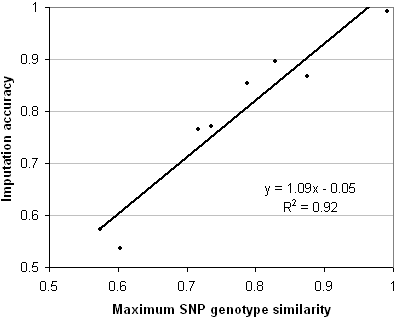

Supplement: Additional file 20 — Dependence of SNP genotype imputation accuracy in a sorghum accession on its maximum similarity to any of the remaining seven accessions. Pairwise similarity was defined on the basis of all SNPs assayed in the panel, as described in the text. [file 1471-2164-12-352-S20.PNG]
